# Urgent surgical management in a patient with bilateral scrotal abscess and necrotizing fasciitis: A case report

**DOI:** 10.1097/MD.0000000000048019

**Published:** 2026-03-13

**Authors:** Jinmeng Hao, Jia Liu, Bo Feng, Zhanping Shi

**Affiliations:** a Department of Urology, People’s Hospital of Yongqing, Yongqing, China.

**Keywords:** case report, debridement, necrotizing fasciitis, scrotal abscess, wound infection

## Abstract

**Rationale:**

Bilateral scrotal abscess complicated by necrotizing fasciitis is a rare but severe clinical emergency that can lead to life-threatening complications such as sepsis and multiorgan failure. Due to its rapid progression and diverse clinical presentation, early diagnosis and treatment are critical to improving patient outcomes.

**Patient concerns:**

A 67-year-old Asian male patient of Chinese nationality presented with bilateral scrotal swelling, pain, and fever. The patient reported severe, persistent scrotal pain accompanied by systemic symptoms, including fever and malaise.

**Diagnoses:**

Physical examination revealed significant scrotal swelling, erythema, and tenderness, with a fluctuant sensation. Laboratory findings showed leukocytosis and elevated C-reactive protein levels, while ultrasound confirmed the presence of bilateral scrotal abscesses and evidence of fascial necrosis.

**Interventions:**

The patient underwent urgent surgical intervention, including bilateral scrotal incision and drainage, and debridement of necrotic tissue.

**Outcomes:**

Postoperatively, the patient was managed with broad-spectrum antibiotics and supportive therapy, leading to gradual improvement and eventual recovery.

**Lessons:**

This case highlights the rarity and urgency of bilateral scrotal abscess complicated by necrotizing fasciitis, emphasizing the importance of heightened clinical suspicion, prompt imaging, and surgical intervention. Early recognition and aggressive treatment are essential for optimizing patient outcomes in such cases.

## 1. Introduction

Bilateral scrotal abscess is a relatively uncommon condition in urological surgery, typically resulting from bacterial infections, with Streptococcus and Staphylococcus species being the most common pathogens.^[1,2]^ The formation of scrotal abscesses often arises from local trauma, ascending infections, or the spread of infections from adjacent structures such as the prostate or epididymis.^[3]^ However, the co-occurrence of bilateral scrotal abscess and necrotizing fasciitis is exceedingly rare and represents a medical emergency requiring immediate intervention to prevent severe complications.^[4]^

Necrotizing fasciitis is a severe soft tissue infection characterized by rapid necrosis of subcutaneous tissue and fascia, often leading to multiorgan failure and high mortality rates.^[5]^ This condition is typically caused by polymicrobial infections, including both Gram-positive and Gram-negative bacteria, as well as anaerobic organisms. In the context of the genitourinary system, necrotizing fasciitis often occurs when localized infections are not promptly controlled or when treatment is delayed.^[6]^ The simultaneous occurrence of bilateral scrotal abscesses and fascial necrosis is an uncommon scenario, potentially linked to specific patient susceptibility factors or the virulence of the infecting pathogens.

While bilateral scrotal abscesses have been documented in the medical literature, cases involving necrotizing fasciitis are exceedingly rare. This uncommon condition presents significant diagnostic and therapeutic challenges, particularly in terms of early recognition and timely intervention. This report aims to provide a detailed account of a rare case of bilateral scrotal abscess complicated by necrotizing fasciitis, analyzing its clinical presentation, diagnostic process, treatment approach, and prognosis. By sharing this case, we hope to enhance clinicians’ awareness of this condition, emphasize the importance of early diagnosis and prompt surgical intervention, and offer insights for the management of similar cases.

## 2. Case report

### 2.1. Patient history

A 67-year-old male patient presented to the urology department with a 4-day history of progressive bilateral scrotal swelling, severe pain, and fever. The patient reported no prior history of similar symptoms or scrotal trauma. He denied recent urological or surgical interventions. His past medical history was notable for type 2 diabetes mellitus, managed with oral medications. He had no known drug allergies or significant family history of similar conditions.

### 2.2. Physical examination

Upon presentation, the patient was in acute distress, with a body temperature of 37.2°C, heart rate of 100 beats/mine, and blood pressure of 140/70 mm Hg.

Upon presentation, the patient exhibited significant clinical findings indicative of bilateral scrotal involvement and systemic infection. A thorough physical examination was conducted, revealing the following specific findings:

(1) Abdominal and groin examination
  - The patient displayed noticeable swelling and tenderness in the left lower abdomen and left inguinal region. This suggested possible lymphatic spread or involvement of the inguinal lymphatics.
  - A crepitus feeling was palpable in the left inguinal area, indicative of subcutaneous gas formation, which is a hallmark of necrotizing fasciitis.
(2) Scrotal examination
  - Both scrotal regions were erythematous, edematous, and tender to palpation. The left scrotum exhibited multiple areas of discoloration, with patches of darkened skin suggesting necrosis.
  - Pronounced crepitus and a fluctuant sensation were palpable in the left scrotum, confirming the presence of deep-seated infection and necrosis. These findings highlighted the severity of the infection and the need for immediate intervention.
(3) Urinary system examination
  - The bladder area was unremarkable, with no evidence of distension or tenderness on palpation. The absence of bladder symptoms, such as frequency or dysuria, suggested that the infection was primarily localized to the scrotal region.
  - The urethral meatus was free of bleeding or discharge, further supporting the absence of primary urethral involvement.

These findings underscored the rapid progression of the infection, involving not only the scrotal region but also extending into the inguinal area. The presence of crepitus and fluctuance provided critical evidence for the diagnosis of bilateral scrotal abscess complicated by necrotizing fasciitis. Such findings are critical for guiding prompt and aggressive surgical management.

### 2.3. Imaging and laboratory findings

Imaging studies revealed significant findings that corroborated the clinical suspicion of bilateral scrotal abscess with necrotizing fasciitis. The ultrasound examination demonstrated the following:

1. Right epididymis: A small cystic lesion was identified in the head of the right epididymis, consistent with an epididymal cyst.
2. Scrotal skin and soft tissues: Significant thickening and swelling of the bilateral scrotal skin were observed, with scattered hyperechoic foci noted in the soft tissues. These findings were suggestive of inflammatory changes and possible abscess formation.

Laboratory and biochemical testing provided further evidence supporting the diagnosis:

(1) Blood glucose: Fasting blood glucose was 17.45 mmol/L (↑), indicating poorly controlled diabetes mellitus, which may have contributed to the patient’s immunocompromised state and susceptibility to infection.
(2) Electrolytes: Sodium level was 131.00 mmol/L (↓), suggesting possible dehydration or systemic inflammatory response.
(3) Inflammatory markers:
  - Interleukin-6 was 822.10 pg/mL (↑), indicating a significant systemic inflammatory response.
  - High-sensitivity C-reactive protein was 120.40 mg/L (↑), further supporting the presence of active inflammation.
  - Coagulation studies: Fibrinogen was 7.55 g/L (↑), suggesting a hypercoagulable state, which may be secondary to the acute inflammatory process.
(4) Complete blood count:
  - White blood cell count: 10.52 × 10^9^/L (↑), indicating leukocytosis.
  - Neutrophils: 9.79 × 10^9^/L (↑), consistent with a bacterial infection.
  - Lymphocytes: 0.54 × 10^9^/L (↓), reflecting a shift in the differential count towards neutrophils, as expected in bacterial infections.
  - Eosinophils: 0.01 × 10^9^/L (↓), which may be secondary to the systemic inflammatory response.

The imaging and laboratory findings collectively supported the clinical diagnosis of bilateral scrotal abscess complicated by necrotizing fasciitis. The elevated inflammatory markers (interleukin-6 and high-sensitivity C-reactive protein) and leukocytosis with a neutrophil predominance indicated a severe bacterial infection. The hyperglycemia and low sodium levels suggested systemic stress and possible dehydration, which are common in severe infections. The elevated fibrinogen level further highlighted the acute phase response to inflammation. The ultrasound findings of bilateral scrotal skin swelling and scattered hyperechoic foci were consistent with the clinical presentation of abscess formation. However, the imaging findings did not fully capture the extent of necrotizing fasciitis, which was later confirmed intraoperatively. These findings underscore the importance of integrating clinical, laboratory, and imaging data to guide the diagnosis and management of complex infections like bilateral scrotal abscess with necrotizing fasciitis.

### 2.4. Surgical procedure

On the day of admission, the patient underwent an emergency scrotal incision and drainage procedure. Under satisfactory general anesthesia, the patient was positioned in a supine position. The surgical field was thoroughly disinfected with iodine-based antiseptic solution. A linear skin incision of approximately 5.0 cm was made at the upper aspect of the left scrotal abscess. Upon incision, approximately 30 mL of aerated, thin purulent exudate and necrotic tissue were observed and evacuated. A second linear incision of about 3.0 cm was made at the lower aspect of the left scrotal abscess to ensure complete drainage.

**Figure 1.**
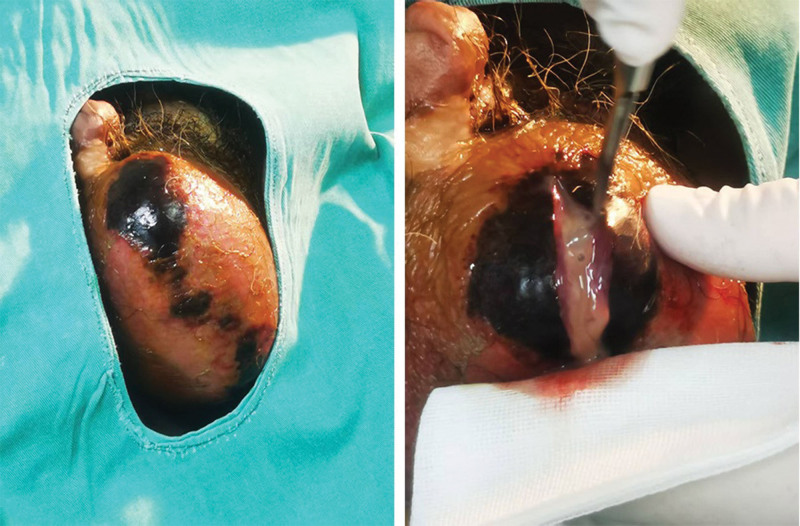
Initial appearance during surgery. (Source: Surgical Procedure section)

Following thorough drainage of pus and necrotic tissue, the surgical wounds were irrigated sequentially with hydrogen peroxide, iodine-based antiseptic solution, and saline. Hemostasis was achieved, and the surgical site was carefully examined to confirm the absence of residual pus or necrotic tissue. A surgical gauze strip was placed in the surgical wound to facilitate drainage, and the area was covered with sterile gauze followed by compression bandaging. The procedure was completed without significant intraoperative bleeding, and the patient was transferred to the recovery room in stable condition. Intraoperative details are illustrated in Figure 1.

This surgical intervention aimed to achieve complete drainage of the abscess, remove necrotic tissue, and control the infection, thereby preventing further progression of the disease. The procedure was deemed successful, with no major complications encountered during the operation.

### 2.5. Postoperative management

The postoperative management of the patient was complex and required a multidisciplinary approach to address the severity and progression of the infection, as well as the underlying diabetes mellitus. The following details the key aspects of the patient’s postoperative care:

1 Immediate postoperative day

The patient was closely monitored in the postanesthesia care unit and subsequently transferred to the surgical ward. Intravenous meropenem was initiated to combat the severe infection. Blood glucose levels were closely monitored, with pre- and post-meal blood glucose readings taken to manage the patient’s poorly controlled diabetes. On the same day, additional incisions and drainage procedures were performed on the left inguinal and lower abdominal regions, with three 1 cm incisions made in the left lower abdomen to facilitate thorough drainage.

At the same time, the endocrine department will conduct consultation on the management of postoperative diabetes. Fasting venous plasma glucose was measured at 21.75 mmol/L, and glycosylated hemoglobin was found to be 11.4%, confirming the diagnosis of diabetes mellitus. Urinalysis revealed ketonuria (1+), leading to the diagnosis of diabetic ketoacidosis. The patient was initiated on low-dose intravenous insulin and fluid therapy to correct the ketoacidosis. Once stabilized, insulin pump therapy was commenced for intensive blood glucose control. The patient’s weight of 73 kg guided the insulin pump settings, with a basal rate of 14 units and premeal boluses of 5 units. The patient was educated on a low-sodium, low-fat diet, regular exercise, and self-monitoring of blood glucose levels. Adjustments to the insulin pump were made based on blood glucose readings, and the patient was closely followed by the urology team.

2 Postoperative days 3 to 5

On postoperative days 3 to 5, the patient continued to exhibit significant swelling and tenderness in the left inguinal region, with crepitus and fluctuance noted. A small amount of purulent discharge was observed from the surgical wounds. The bladder area remained unremarkable, with no evidence of distension or tenderness. The right scrotum showed improvement in erythema and swelling, while the left scrotum demonstrated ongoing erythema, with areas of necrosis and exudate. On postoperative day 5, the surgical wounds were reexamined and cleaned with hydrogen peroxide and iodine-based antiseptic solution. Despite these measures, the left scrotum continued to show areas of necrosis.

3 Postoperative days 6 to 10

On postoperative day 6, the extent of necrosis and purulent drainage in the scrotal region necessitated the extension of the scrotal incisions to ensure complete drainage. By postoperative day 7, the infection had spread to the abdominal wall, requiring the extension of the 3 existing abdominal incisions and the addition of 2 new incisions with drainage tubes. On postoperative day 10, the scrotal incisions were connected to allow for better drainage, and the testes were temporarily exteriorized to facilitate wound healing.

4 Postoperative days 11 to 15

During this period, the abdominal cavity drainage decreased to approximately 100 to 120 mL/d. A CT scan performed on that day revealed that the infection in the left abdominal wall and bilateral scrotum had improved compared to the initial imaging. However, new findings of infection in the left short external rotator muscles, adductor magnus, and gluteus maximus muscles were noted, requiring continued monitoring and follow-up.

5 Postoperative days 16 to 20

Abdominal drainage decreased further to 80 to 100 mL/d. On postoperative day 20, the patient developed a new infection in the left popliteal fossa, with significant purulent drainage (approximately 500 mL) and necrotic tissue. Two linear incisions of 3.0 cm were made in the upper and lower aspects of the popliteal fossa to facilitate drainage. The wound was irrigated with hydrogen peroxide, iodine-based antiseptic solution, and saline, and a drainage tube was placed. The surgical site was covered with sterile gauze and compression bandaging.

6 Postoperative says 21 to 28

From postoperative days 21 to 28, the patient’s condition continued to improve. Abdominal drainage decreased to 20 to 50 mL/d, while left lower extremity drainage stabilized at approximately 100 mL/d. The scrotal wounds showed progressive healing, with reduced erythema and exudate.

7 Postoperative days 29 to 50

By postoperative day 45, the abdominal wounds had fully healed. On postoperative day 50, the scrotal wounds were closed primarily, and sutures were removed upon healing. The patient’s blood glucose levels were well-controlled throughout this period, with continued insulin pump therapy and adherence to dietary and exercise recommendations.

**Figure 2.**
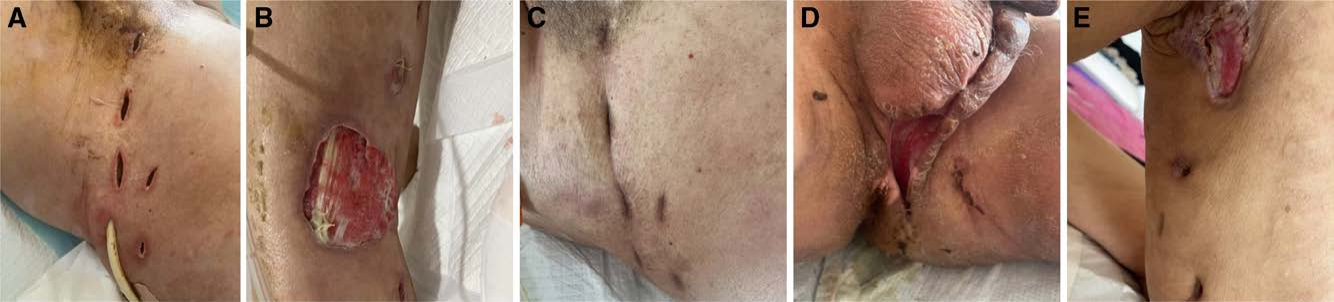
Postoperative incision recovery of the patient. (A) Depicts the condition on the 7th day post-surgery. (B) Illustrates the state on the 22nd day following surgery. (C) Demonstrates the status on the 45th day post-surgery. (D) Culminates in the situation on the 50th day following surgery. (E) Portrays the outcome following the removal of the perineal cord.

The postoperative management of this patient was marked by the complexity and progression of the infection, requiring multiple surgical interventions, extensive drainage, and intensive antimicrobial therapy. The involvement of the Endocrinology Department was critical in managing the patient’s diabetes mellitus and ketoacidosis, which played a significant role in his immunocompromised state and susceptibility to severe infection. Through a multidisciplinary approach, aggressive surgical management, and meticulous wound care, the patient achieved a favorable outcome with complete resolution of the infection and successful wound healing. This case underscores the importance of early recognition, timely surgical intervention, and comprehensive postoperative care in managing severe infections such as bilateral scrotal abscess complicated by necrotizing fasciitis. The recovery of the patient’s scrotum is shown in Figure 2.

## 3. Discussion

The present case represents a rare and severe clinical entity, bilateral scrotal abscess complicated by necrotizing fasciitis, which is uncommonly encountered in clinical practice. The patient presented with advanced infection, involving not only the scrotal region but also extending to the inguinal, abdominal, popliteal, and proximal femoral regions. The simultaneous occurrence of bilateral scrotal abscess and extensive necrotizing fasciitis underscores the aggressive nature of this condition, which may rapidly progress to systemic sepsis and multiple organ failure if not promptly managed.^[7,8]^ To the best of our knowledge, such a comprehensive and extensive involvement of the genitourinary system is seldom reported in the literature, highlighting the uniqueness of this case.

The patient’s underlying type 2 diabetes mellitus and ketonemia played significant roles in predisposing him to this severe infection.^[9]^ Diabetes mellitus is well-documented to impair immune function and wound healing, thereby increasing the risk of bacterial infections. In this case, the uncontrolled hyperglycemia and ketonemia likely contributed to the rapid progression and severity of the infection. The presence of ketonemia may also have exacerbated systemic inflammatory response, further complicating the clinical course. This case serves as a reminder of the heightened susceptibility to severe infections in diabetic patients and the importance of tight glycemic control in preventing such complications.

Prompt recognition and aggressive surgical intervention were critical in this patient’s survival and recovery. The multidisciplinary approach involving urological surgery, anesthesiology, and endocrinology was pivotal in managing both the infection and the underlying diabetic ketoacidosis.^[10]^ Early imaging studies and laboratory analyses facilitated the accurate diagnosis, enabling immediate surgical debridement and drainage. The ability to swiftly identify and address the infection source was instrumental in preventing further systemic deterioration and multiorgan failure.

The surgical management of this case was marked by its complexity and extent. Multiple incisions and drainage procedures were performed across various anatomical regions, reflecting the widespread nature of the infection. The need for repeated debridements and the eventual exteriorization of the testes underscored the severity of the necrotizing process. The collaboration with the endocrinology department was essential in managing the patient’s diabetes, ensuring optimal conditions for wound healing and recovery. This interdisciplinary approach highlights the importance of coordinated care in managing complex, multisystem infections.

This case report provides several key insights for clinical practice. Clinicians should maintain a high index of suspicion for necrotizing fasciitis in male patients presenting with scrotal swelling and pain, especially in those with underlying diabetes mellitus. Early surgical exploration and debridement are imperative in preventing the progression of localized infections to life-threatening conditions.^[11,12]^ Effective management of complex infections necessitates collaboration across specialties, including urology, anesthesiology, and endocrinology. Optimal management of diabetes and glycemic control is crucial in reducing the risk of severe infections and improving outcomes.

In conclusion, this case report illustrates the severe complications that can arise from bilateral scrotal abscesses complicated by necrotizing fasciitis. Timely diagnosis, aggressive surgical intervention, and comprehensive multidisciplinary care were instrumental in achieving a favorable outcome. This case emphasizes the importance of early recognition, rapid response, and coordinated care in managing rare and severe infections, particularly in patients with underlying chronic conditions like diabetes mellitus. Further research is needed to better understand these complex conditions and improve therapeutic strategies for similar cases in the future.

## Author contributions

**Conceptualization:** Jinmeng Hao.

**Data curation:** Jinmeng Hao, Jia Liu, Bo Feng, Zhanping Shi.

**Methodology:** Jinmeng Hao, Jia Liu, Bo Feng, Zhanping Shi.

**Writing – original draft:** Jinmeng Hao.

**Writing – review & editing:** Jinmeng Hao, Jia Liu, Bo Feng, Zhanping Shi.
